# A Comparison between the i-gel® and air-Q® Supraglottic Airway Devices Used for the Patients Undergoing General Anesthesia with Muscle Relaxation

**DOI:** 10.1155/2018/5202957

**Published:** 2018-11-18

**Authors:** Nilofar Massoudi, Mohammad Fathi, Navid Nooraei, Alireza Salehi

**Affiliations:** ^1^Clinical Research and Development Unit at Shahid Modarres Hospital, Department of Anaesthesiology, Shahid Beheshti University of Medical Sciences, Tehran, Iran; ^2^Anesthesiology Research Center, Department of Anesthesiology, Shahid Beheshti University of Medical Sciences, Tehran, Iran

## Abstract

**Objectives:**

The aim of the present study was to compare two supraglottic airway (SGA) devices (i.e., the i-gel® © Intersurgical Ltd and air-Q® (Reusable) Cookgas company) in terms of the insertion time, amount of leak during ventilation with maximum positive pressure, and postoperative complications in patients referring to Modarres Hospital in Tehran.

**Method:**

The present double-blind clinical trial was performed on 60 patients undergoing elective surgeries that required general anesthesia with muscle relaxation. Patients were randomly assigned to either i-gel® (*n* = 30) or Air-Q® (*n* = 30) groups.

**Results:**

The mean age, body mass index, duration of surgery, duration of anesthesia, and gender ratio were not significantly different between the two groups. Mean ± SD values of the SGA devices' insertion time (in seconds) in the air-Q® and i-gel® groups were 4.87 ± 1.6 and 6.80 ± 1.2, respectively (P < 0.001). The mean OLP in the Air-Q® group was significantly higher than that of the i-gel® group (35.9 ± 9.6 versus 24.8 ± 3.7, p < 0.001). The frequency of complications occurred after the supraglottic airway insertion was higher in the i-gel® group. However, only in terms of sore throat, the difference between the two groups was statistically significant: 6 (20%) had sore throat (P = 0.024) in the i-gel groups, but in in the Air-Q® groups no one had this side effect after surgery.

**Conclusion:**

It was concluded that the Air-Q® supraglottic airway was placed faster and easier with fewer complications than the i-gel in general anesthesia with muscle relaxation. The frequency of the occurrence of all three complications, cough, sore throat, and blood, on the cuff (6 (20%) was higher in the i-gel group than that in the air-Q® group (cough3 (10%), sore throat 0 (0%), and blood on the cuff 3 (10%) (P < 0.05).

## 1. Introduction

One of the most important challenge of anesthesia is airway managment [1]. Establishing an airway is considered as one of the most important anesthesiologists' skills, so being not able to establish an airway may cause disastrous consequences [2].

After the old anesthesia techniques that resulted in adverse effects of hypoxia caused by airway obstruction, tracheal intubation was considered as a special and great evolution in establishing an airway in anesthetic conditions by anesthetists and other specialists. So, for a long time, there was not much interest in other airway devices [1]. This attitude gradually began to change with the emergence of supraglottic airway devices [1]. Supraglottic airway devices are a family of medical devices that facilitate ventilation and oxygenation without the need for endotracheal intubation [2]. The emergence of laryngeal mask (LMA) as the first supraglottic airway device [3] was a turning point in airway management [1].

Major complications include pharyngeal rupture, pneumomediastinum, mediastinitis, or arytenoid dislocation. Mild short-lasting side effects of the devices have significantly higher incidence than serious complications and involve postoperative sore throat, dysphagia, pain on swallowing, or hoarseness. Devices may have harmful effect on cervical mucosa or vasculature contingent on their cuff volume and pressure [4].

With the advent of newer supraglottic airway devices, one of the important issues is to compare them in terms of safety and efficacy [5]. In the current study, two of these devices, namely, the i-gel and air-Q, were compared. The i-gel airway (i-gel, Intersurgical Ltd.) is a supraglottic airway device that is made up of a thermoplastic elastomer that looks like a soft gel. It is designed so that it can seal the perilaryngeal and hypopharyngeal structures. This device does not have an inflatable cuff, but it is equipped with a port for gastric tube insertion. Among the benefits of this device, convenient insertion, postinsertion stability, and a minimum risk of tissue compression can be mentioned [6]. The air-Q airway is also a polyvinyl chloride supraglottic airway device. This device has a supraglottic oval-shaped hypercurved part, which, alien with a tracheal tube, facilitates the insertion of the device [6]. This device has a tube with a large flexible cuff at the bottom and is designed to be fitted in the hypopharynx [7]. The tip of the cuff is designed to prevent blockage of the device's lumen by the epiglottis. On the other hand, having no diaphragm strips makes it possible for the endotracheal tube to pass through this device with no obstacle. Thus, with the help of this supraglottic airway device, trachea can be intubated without the need for direct laryngoscopy with its known dangers [7]. Considering the fact that there is a dearth of study on comparing the safety and efficacy of these two devices, this study attempted to compare the i-gel and air-Q devices in terms of the insertion time, amount of leak during maximum positive-pressure ventilation, and postoperative complications including sore throat, cough, and trauma in the patients referring to Modarres Hospital in Tehran for undergoing elective surgeries.

## 2. Procedure

This clinical trial was performed on 60 patients with ASA I-II who referred to Shahid Modarres Hospital in Tehran and were candidates for minor elective surgeries under general anesthesia with muscle relaxation and controlled ventilation. The inclusion criteria included (1) being candidate for minor elective surgeries under general anesthesia with muscle relaxation, while ventilation was done by using a ventilator, (2) ASA Class I-II, (3) age of 18-70 years, and (4) willingness to consent to participation in the described study. The exclusion criteria contained (1) having advanced cardiovascular problems (ASA Class III-IV), (2) duration of surgery longer than 2 hours, (3) over 70 or under 18 years of age, (4) lack of consent to participate in the study, and (5) emergency surgeries. Written informed consent was obtained from all subjects and after that their demographic information including age, gender, and body mass index was initially recorded. Then, the patients were randomly allocated by consuming computer-generated tables of random numbers into two groups: i-gel (n = 30) or air-Q (n = 30). The present study was designed as a double‐blind, randomized, parallel‐group study. We determine an anesthesiologist who inserted supraglottic device; the anesthesiologist who placed the device was not involved in the study and neither was the technician of anesthesia collecting data, so the author did not know anything about groups of patients' analysis information. Prior to anesthesia induction, an 18-gauge catheter for the administration of fluids and medications was inserted preoperatively. After anesthesia induction with propofol (2.5 mg/kg) and atracurium (0.5 mg/kg), a supraglottic airway device was inserted; the tries for laryngeal mask insertion were taking place after achievement of anesthesia and seeing loss of lash reflex and the jaw relaxation. During the insertion, the anesthetic technician measured the time of insertion of the device, and after the device was fitted, the expiratory valve was fastened at a gas flow of 3 L/min and airway pressure at which the gas leakage was sensed was documented. Oropharyngeal leak pressure was measured with the flow rate of 3 L/min. And then the patient was subjected to controlled ventilation with TV = 5-6 CC/kg, rate = 12, and Pmax = 25. For maintenance of anesthesia, sevoflurane 2.5-1.6 MAC, O2 100%, and atracurium 10 mg were repeated every 20 min. Mean arterial pressure (MAP), heart rate, and ETCO_2_ were measured and recorded at four different time intervals (every 15 minutes after beginning surgery) for all patients at the same time. After the completion of the surgery, the airway device was checked for blood staining. The duration of surgery and duration of anesthesia were recorded for each patient and the patient was asked whether he or she had a feeling of sore throat and cough or not in the postanesthesia care unit.

## 3. Ethical Considerations

The study (date: 5/21/2017, at the 37th meeting of the committee) was approved by a biomedical research ethics committee by code IR.SBMU.RETECH.REC.1396.129 and was registered in IRCT (Iranian Registry Clinical Trial) by code IRCT2016081512203N8.

## 4. Analysis

With a test power of 95%, the type 1 error of 0.05, and the mean ± standard deviation OLP of the air-Q and i-gel groups (i.e., 25.3 ± 9 and 21.4 ± 5.2, resp.), the sample size was 30 in each group.

Quantitative variables were represented as mean ± SD and qualitative variables were represented as number (percentage). The normality of the quantitative variables was verified using the Shapiro-Wilk test and normal quantile plot. Due to the fact that all the quantitative variables were abnormally distributed, a comparison was made between the two groups in terms of quantitative variables using the Mann–Whitney test and in terms of qualitative variables using Pearson's chi-square or Fisher's exact tests. All hypothesis tests were performed on a 2-sided basis. The significance level in all tests was 0.05.

## 5. Results

60 patients who were candidates for minor elective surgeries under general anesthesia with muscle relaxation and under ventilation with a ventilator in Shahid Modarres Hospital were eligible for inclusion in this study. The patients were randomly assigned to the air-Q and i-gel groups. None of the quantitative variables had normal distribution; therefore, the comparison between the two groups in terms of all quantitative variables was performed using the Mann–Whitney test.

In Table 1, the primary characteristics of patients are presented for both groups. As it can be observed, the mean age, body mass index, surgery duration, anesthesia duration, and gender ratio were not significantly different between the two groups.

**Figure 1:**
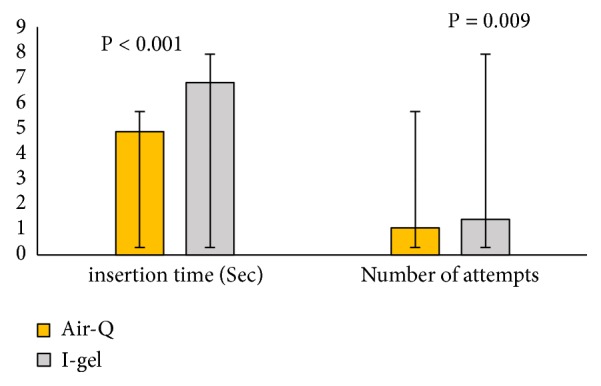
Mean insertion time (in seconds) and mean number of attempts for insertion in the i-gel and air-Q groups.

The mean ± SD of SGA insertion times (in seconds) in the air-Q and i-gel groups were 4.87 ± 1.6 and 6.80 ± 1.2, respectively. The results of the Mann–Whitney test showed that the insertion time in the air-Q group was significantly lower (Figure 1). The mean number of attempts for insertion was also significantly lower in the air-Q group than in the i-gel group (p = 0.009, 1.1 ± 0.2 versus 1.4 ± 0.6) (Figure 1).

**Figure 2:**
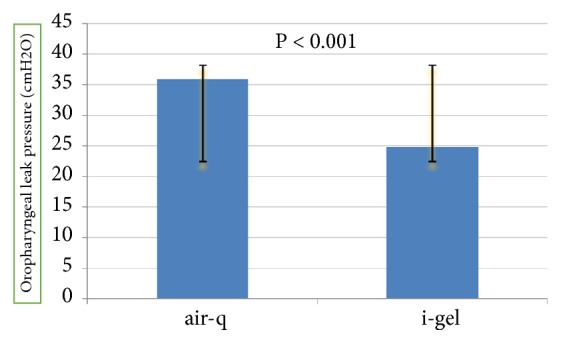
Mean oropharyngeal leak pressure (cmH_2_O) in the i-gel and air-Q groups.

The mean OLP (in cmH_2_O) in the air-Q group was significantly higher than that in the i-gel group (35.9 ± 9.6 versus 24.8 ± 3.7, p < 0.001) (Figure 2).

Hemodynamic parameters including the end-tidal carbon dioxide (ETCO_2_) concentration (in mmHG), MAP (in mmHG), and heart rate (HR) of each patient were measured at four different time intervals. The results of these measurements are presented in Table 2. The values for all of the three variables are depicted in the table.

**Figure 3:**
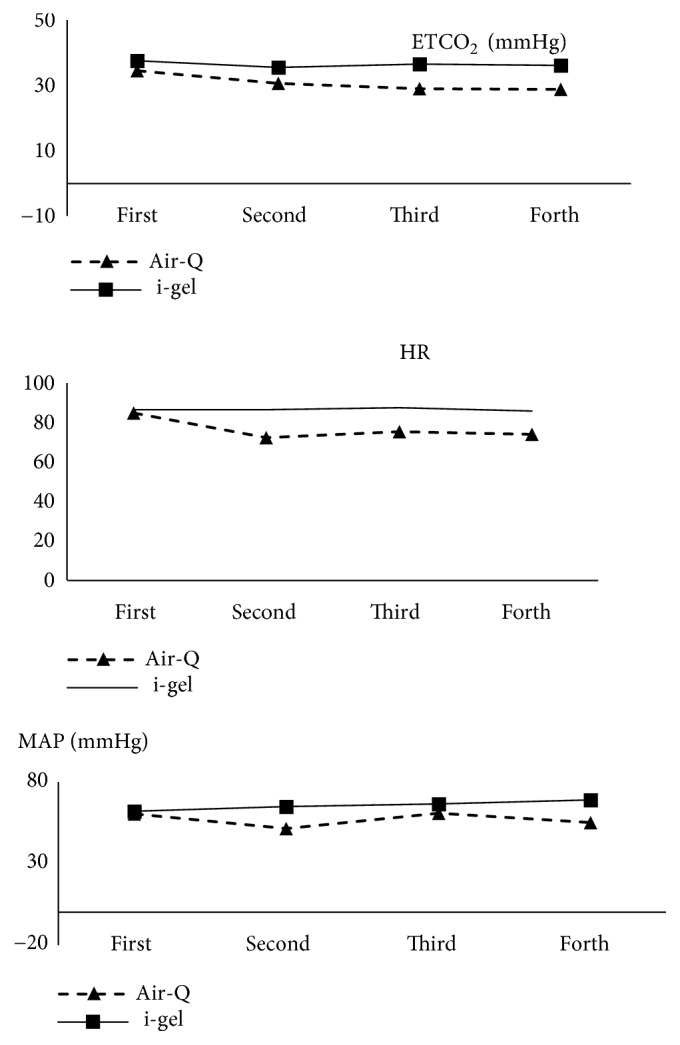
Trends of hemodynamic parameters in the i-gel and air-Q groups.

Time intervals in the air-Q group were lower than those in the i-gel group. These differences were statistically significant for ETCO2; nevertheless, mean blood pressure and heart rate, except for the first-time interval, in three other time intervals were significant.  Figure 3 shows the status of changing these parameters over time. As can be seen, the variations that occurred in the air-Q group were more significant than those of the i-gel group. To evaluate the significance of the changes occurring in the values of these variables at different time intervals, the Friedman test was used. The results of this test are presented in Table 2. As you can see, the changes in the values of all three variables were statistically significant in the air-Q group, whereas in the i-gel® group, only changes in the value of the MAP variable were statistically significant.

The frequency of complications following the placement of the supraglottic airway devices, including cough, sore throat, and blood on the cuff, is presented in Table 3. The frequency of the occurrence of all three complications was higher in the i-gel group than in the air-Q group. However, only in case of sore throat was the difference between the two groups statistically significant, where 6 (20%) had sore throat (p = 0.024) in the i-gel group, but in in the air-Q group no one had this side effect after surgery.

## 6. Discussion

The current clinical trial was an endeavor to compare two supraglottic airway devices including the air-Q and i-gel devices in terms of insertion time, OLP, some hemodynamic characteristics, and postoperative complications [4].

The findings of this study showed that the mean insertion time in the air-Q group was significantly lower than that in the i-gel group. Also, the number of attempts for insertion in the air-Q group was significantly lower than that of the i-gel group. This finding was also asserted in a study by Jagannathan et al [5]. In their study, the mean i-gel insertion time was significantly longer than that of air-Q and the mean number of attempts for insertion was also significantly lower in the air-Q group than in the i-gel group (p = 0.009, 1.1 ± 0.2 versus 1.4 ± 0.6) (Figure 1).

In a comprehensive meta-analysis, in which i-gel was compared with other supraglottic airway devices, it was shown that although i-gel could be placed more quickly than some other supraglottic airway devices, the time it took to be inserted was longer than that of the air-Q and LMA Supreme Aura-i devices [8]. In a clinical trial performed on 100 patients who were candidates for general anesthesia, the success rate of blind tracheal intubation in the air-Q group was 82% (41.50) and for the i-gel group it was 54% (27.50%) (p value = 0.003) [9]. According to Komasava et al. [10], the curved anatomical structure of air-Q plays a role in its easier insertion. Kohama et al. [11] also stated that, during infant chest compression, air-Q was inserted in shorter time than i-gel. They believed that the straight design and the anatomical shape of i-gel could lead to an interruption in its placement in the pharyngeal space during chest compression.

However, muscle relaxation was performed to assist ProSeal insertion technique by facilitating greater successful insertion rates, higher sealing pressure, minor leakage amount, and lower personal force when inserted in anesthetized patients [12].

Oropharyngeal leak pressure (OLP) as an indicator of the efficiency of supraglottic airway devices is the amount of pressure in the airway in which the air leak occurs around the supraglottic airway devices [13]. In this study, the mean OLP in the air-Q group was 11.10 cmH_2_O and was significantly greater than that of the i-gel group. In a study by Damdoran et al. [14], the mean OLP in the air-Q group was 2.3 cmH_2_O higher than that of the i-gel group and was 1.3 cmH_2_O higher than that of the LMA Supreme group; however, there was no significant difference between the three groups in this study [14]. In some other studies, the mean OLP in the i-gel group was higher than that of the air-Q group [8, 13]. On the other hand, the mean OLP in the i-gel group in the present study was similar to that found in other studies [13, 15]. OLP is one of the safety indicators of supraglottic devices [15].

In some other studies, muscle relaxant can diminish the pressure of oropharyngeal muscles and avoid dislocation of FLMA follow-on from contraction of oropharyngeal muscles; it can recover the obedience of thorax and conserve a low airway pressure; it can inhibit spontaneous ventilation and patient-ventilator asynchrony. All this strength is reserved for less air leakage throughout the surgery [16]. Considering the lowest mean insertion time, the number of attempts for insertion, and lower complications associated with the insertion of air-Q, other safety indicators of supraglottic devices in the present study were satisfied in the air-Q group compared with the i-gel group; it can be concluded that the higher mean OLP in the air-Q group, in accordance with other above-mentioned indicators, confirmed the higher safety of air-Q compared with i-gel in the present study. On the other hand, according to a meta-analysis, the reported OLP for a supraglottic airway device depended on numerous factors such as cuff pressure level, leak detection method, use of muscle relaxant, and type of ventilation (controlled versus spontaneous respiration) [17]. Finally, it should be mentioned that, according to Brimacombe et al. [18], the optimal OLP pressure was greater than 10 cmH_2_O, so it can be ensured that both devices in the current study could successfully protect the airway from oropharyngeal secretions.

The hemodynamic parameters compared between the air-Q and i-gel groups were HR, MAP (mmHg), and EtCO_2_ (mmHg). The mean of these three parameters was significantly higher in the i-gel group than in the air-Q group most of the time and the change rates of these parameters in the i-gel group were less than those of the air-Q group. Jindal et al. [19] also observed that changes in hemodynamic parameters following the use of i-gel were the lowest compared with those of LMA and SLIPA. Atef et al. [20] reported the change rate of hemodynamic parameters including HR, SBP, and DBP to be 0% after the insertion of i-gel. In a clinical trial conducted by Bhandari et al. similar to the current study [6], the mean HR in all three steps (i.e., before induction, after induction, and after successful insertion) in the i-gel group was higher than that of the air-Q group, but only in the third stage was this difference statistically significant. Generally, it can be said that although hemodynamic changes that occurred in patients in both groups were desirable, i-gel was more successful than air-Q in terms of efficacy on hemodynamic parameters.

The patients were assessed for the complications of the devices studied such as blood on the cuff after removal of the device and cough and sore throat in the postanesthesia care unit. In this case, the results showed that the frequency of all three of these complications in the i-gel group was greater than that of the air-Q group. In a study done by Kim et al. [13], the number of patients with vomiting and coughing was higher in the i-gel group than in the air-Q group, and, vice versa, the amount of blood stain on the device in the air-Q group was higher than that of the i-gel group. Jagannathan et al [21]. Inadvertent extubation and toughness in managing the tracheal tube were higher in the i-gel® group than in the air-Q® group. In a clinical trial of 60 children, the incidence of complications includes the following: bleeding from soft tissues, the presence of blood on the cuff after exiting, and laryngospasm in the i-gel® group was higher than that in the air-Q® group, but the difference was not statistically significant. Considering that, in the present study, the number of attempts for inserting the device was significantly higher in the i-gel group than in the air-Q group, it can be concluded that the incidence of more complications in the i-gel group could be associated with more effort for inserting the device. The frequency of these complications in the i-gel group was similar to that reported in other studies [6]. The representative incidence of blood on i-gel at removal is between 4% and 13% [4] but has been described to be as high as 20%, although in novice operators [22]. The AuraOnce laryngeal mask was related to a very low (2%) incidence of blood staining after its removal [23] but reached 10% in another study.

## 7. Limitations

Incidents principal to main unfavorable outcomes counted in regurgitation (and aspiration), air leaks, trauma, and device displacement. Even though SGA rehearsal has not been designated for parturient patients, morbidly obese patients, and patients in the prone or lithotomy position, the clinician must consider that these consumptions often drive away from the producers' references and may place both patient and clinician in trouble [24].

So that is why in full stomach patients or patients with morbid obesity or prone position could not use supraglottic airway device.

## 8. Conclusion

The air-Q airway device was fitted more quickly and easily than the i-gel device. The mean OLP was also higher in the air-Q group than in the i-gel group, which could improve airway sealing, and the frequency of complications following the removal of the device including cough, sore throat, and blood on the cuff was lower in the air-Q group than in the i-gel group. Our study recommended that air-Q is a suitable device for airway management in patients who need general anesthesia. Easy application and low complications of the device could present air-Q as a suitable replacement of i-gel.

## Figures and Tables

**Table 1:** Characteristics of the i-gel and air-Q groups.

	**i-gel ** **(n=30)**	**air-Q ** **(n=30)**	**p value** ^**a**^
**Age (years)**	37.30 ±15.8	33.73 ± 11.4	0.549
**Gender (female)**	14 (46.7%)	13 (43.3%)	0.795^b^
**BMI (kg/m** ^**2**^ **)**	21.40 ± 1.6	22.23 ± 1.9	0.069
**Operation duration (h)**	2.21 ± 0.6	2.21 ± 0.6	1.00
**Anesthesia duration (h)**	1.63 ± 0.5	1.63 ± 0.5	1.00

^a^By the Mann–Whitney test; ^b^by Pearson's chi-square.

**Table 2:** Comparison of hemodynamic characteristics between the i-gel and air-Q groups.

**Variables**		**air-Q** **(n=30)**	**i-gel** **(n=30)**	**p value** ^**a**^
**ETCO2 (mmHG)**	First	34.50 ±3.3	37.63 ± 4.8	0.009
	Second	30.57 ± 2.6	35.43 ± 3.5	< 0.001
	Third	28.90 ± 2.7	36.43 ± 4.2	< 0.001
	Forth	28.80 ± 2.8	36.13 ± 3.8	< 0.001
	Friedman test results (Chi^2^, p value)	(47.02, < 0.001)	(4.81, 0.187)	.

**MAP (mmHG)**	First	60.30 ± 6.7	62.03 ± 7.2	0.513
	Second	51.67 ± 5.0	65.17 ± 5.9	< 0.001
	Third	60.97 ± 5.1	66.80 ± 3.4	< 0.001
	Forth	55.27 ± 7.7	69.03 ± 6.8	< 0.001
	Friedman test results (Chi^2^, p value)	(43.08, < 0.001)	(14.29, 0.003)	.

**HR**	First	85.03 ± 14.0	86.67 ± 13.7	0.587
	Second	72.47 ± 9.3	86.50 ± 11.2	< 0.001
	Third	75.57 ± 6.1	87.70 ± 8.1	< 0.001
	Forth	74.23 ± 7.5	85.80 ± 7.2	< 0.001
	Friedman test results (Chi^2^, p value)	(21.44, < 0.001)	(2.59, 0.459)	.

^a^By the Mann–Whitney test.

**Table 3:** Comparison of adverse events following the insertion of supraglottic airway devices in the i-gel and air-Q groups.

	**air-Q** **(n=30)**	**i-gel** **(n=30)**	**p value** ^**a**^
**Bloody cuff (yes)**	3 (10%)	6 (20%)	0.472
**Sore throat (yes) **	0 (0%)	6 (20%)	0.024
**Cough (yes)**	3 (10%)	6 (20%)	0.472

^a^By Fisher's exact test.

## Data Availability

The data used to support the findings of this study are available from the corresponding author upon request.
